# Phenotypic trait variation measured on European genetic trials of *Fagus sylvatica* L

**DOI:** 10.1038/sdata.2018.149

**Published:** 2018-07-31

**Authors:** T. Matthew Robson, Marta Benito Garzón, BeechCOSTe52 database consortium, Ricardo Alia Miranda, Diana Barba Egido, Saša Bogdan, Attila Borovics, Gregor Božič, Oliver Brendel, Jo Clark, Sven M.G De Vries, Ivan I Delehan, Alexis Ducousso, Bruno Fady, John Fennessy, Manfred Forstreuter, Josef Frýdl, Thomas Geburek, Dušan Gömöry, Maria Hauke-Kowalska, Gerhard Huber, Juan-Ignacio Ibañez, Lucia Ioniţă, Mladen Ivankovič, Jon Kehlet Hansen, Anikó Kóczán- Horváth, Hojka Kraigher, Steve Lee, Mirko Liesebach, Csaba Mátyás, Patrick Mertens, Hans-Jakob Muhs, Petr Novotný, Gheorghe Parnuţa, Ladislav Paule, Alvaro Picardo, Ervin Rasztovics, Martin Rogge, Lars-Göran Stener, Małgorzata Sułkowska, Otmar Urban, Georg Von Wuehlisch, Giovanni G Vendramin, Cristina Vettori, Wojciech Wesoły

**Affiliations:** 1 Viikki Plant Science Centre (ViPS), Faculty of Biological and Environmental Sciences, University of Helsinki, Helsinki, P.O. Box 65, 00014, Finland; 2 UMR 1202 Biodiversité Gènes & Communautés, INRA, Université Bordeaux, Bâtiment B2llée G St Hilaire, CS 50023, 33615 Pessac Cedex, France; 3 Insitituto Nacional de Investigacion y Tecnologia Agraria y Alimentaria, INIA, Madrid, Spain.; 4 University of Zagreb, Faculty of Forestry, Zagreb, Croatia.; 5 Hungarian Forest Research Institute, Sárvár, Hungary.; 6 Slovenian Forestry Institute, Ljubljana, Slovenia.; 7 UMR EEF, INRA, Université de Lorraine, F-54280 Champenoux, France.; 8 Earth Trust, Little Wittenham, Abingdon, Oxon OX14 4QZ, UK.; 9 Alterra Environmental Research, Wageningen University and Research Centre, Wageningen, The Netherlands.; 10 Ukrainian State University of Forestry and Wood Technology, Faculty of Forestry, Lviv, Ukraine.; 11 BIOGECO, INRA, Université de Bordeaux, 33610 Cestas, France.; 12 URFM, INRA, Domaine St. Paul 84914, Avignon, France.; 13 COFORD-National Council for Forest Research and Development, Dublin, Ireland.; 14 FU Berlin BW, Germany.; 15 Forestry and Game Management Research Institute, Strnady 136, 25202, Jíloviště. Czech Republic.; 16 Federal Forest Research Centre, Vienna, Austria.; 17 Faculty of Forestry, Technical University in Zvolen, 96053 Zvolen, Slovakia.; 18 Poznań University of Life Sciences, Department of Forest Silviculture, Poznan, Poland.; 19 Bavarian Office for Forest Seeding and Planting (ASP), Teisendorf, Germany.; 20 Forest Service, La Rioja, Logroño, Spain.; 21 Academy of Agriculture and Silviculture, Bucharest, Romania.; 22 Croatian Forest Research Institute, Division of Genetics, Forest Tree Breeding and seed husbandry, Jastrebarsko, Croatia.; 23 University of Copenhagen, Department of Geosciences and Natural Resource Management, Section for Forest, Nature and Biomass, Copenhagen, Denmark.; 24 University Sopron, Inst. Env. and Geo Sci, Sopron pob 132, Hungary.; 25 Forest Research, Northern Research Station, Roslin, Midlothian, UK.; 26 Thünen Institute of Forest Genetics, Grosshansdorf, Germany.; 27 Station de Recherches Forestières, Gembloux, Belgium.; 28 Directorate of Environment, Junta de Castilla y León, Valladolid, Spain.; 29 Landesbetrieb Wald und Holz NRW, Arnsberg, Germany.; 30 The Forestry Research Institute of Sweden, Ekebo, Sweden.; 31 Forest Research Institute, Warsaw, Poland.; 32 CZECHGLOBE Global Change Research Institute CAS, Brno, Czech Republic.; 33 Institute of Biosciences and BioResources, National Research Council, Florence, Italy.

**Keywords:** Forestry, Natural variation in plants, Biogeography, Forest ecology, Ecological genetics

## Abstract

We present *BeechCOSTe52*; a database of European beech (*Fagus sylvatica*) phenotypic measurements for several traits related to fitness measured in genetic trials planted across Europe. The dataset was compiled and harmonized during the COST-Action E52 (2006–2010), and subsequently cross-validated to ensure consistency of measurement data among trials and provenances. Phenotypic traits (height, diameter at breast height, basal diameter, mortality, phenology of spring bud burst and autumn–leaf discoloration) were recorded in 38 trial sites where 217 provenances covering the entire distribution of European beech were established in two consecutive series (1993/95 and 1996/98). The recorded data refer to 862,095 measurements of the same trees aged from 2 to 15 years old over multiple years. This dataset captures the considerable genetic and phenotypic intra-specific variation present in European beech and should be of interest to researchers from several disciplines including quantitative genetics, ecology, biogeography, macroecology, adaptive management of forests and bioeconomy.

## Background & Summary

Currently climate change threatens to outpace forest migration, making trees’ survival dependent on their intrinsic potential to adapt and acclimatise to new climatic conditions. Judging the extent of trees’ capacity to cope successfully with new conditions requires knowledge of their tolerance limits; something that is hard to study and to-date cannot be estimated with confidence for many tree species. The long life-span of forest trees means that we might expect to see a lag in their local adaptation to climate; it also makes experiments requiring comparisons across multiple generations difficult. Genetic trials planted for commercial purposes over recent decades probably hold the greatest potential to provide those data that will allow us to understand adaptive processes and the acclimation of trees to new climates. While analyses of individual trials comparing several provenances can inform us of those differences among populations that are shaped by genetics, only information gleaned from multiple trials covering an entire species range allow us to assess the relative importance of genetics and phenotypic plasticity, and how the two interact, for species survival under a changing climate^1–4^.

European beech (*Fagus sylvatica* L.) is an important tree species for forestry and culturally. It covers roughly 14-million ha of forested land, ranging from Mediterranean to temperate ecosystems (http://www.euforgen.org). This wide ecological amplitude makes beech a good reference species for large-scale studies of plastic and adaptive responses in its fitness-related traits to climate change over its full distribution range.

**Figure 1:**
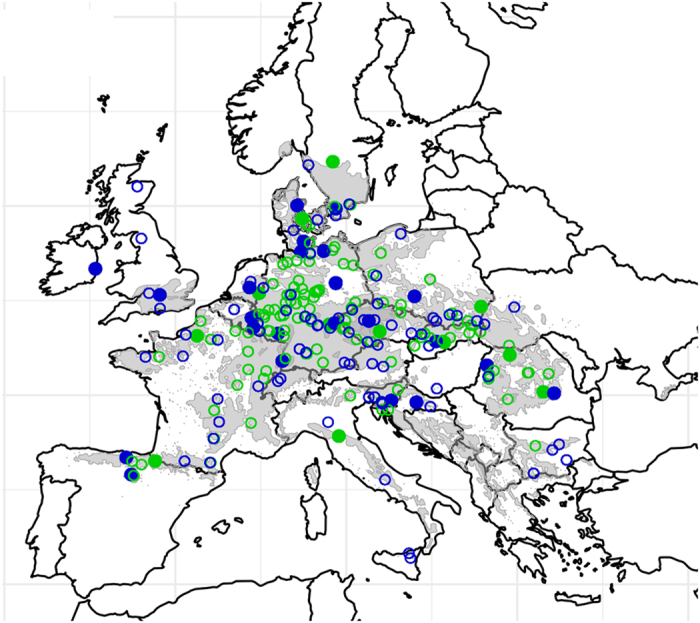
Location of the genetic trials and provenances tested across Europe. The distribution of *Fagus sylvatica* is reproduced in grey from EUFORGEN http://www.euforgen.org/distribution-maps/. Plotted symbols represent trials established in 1995 (green solid circles) and 1998 (blue solid circles) and the respective provenances tested in series from 1995 (green empty circles) and 1998 (blue empty circles). Only locations and provenances for which phenotypic traits have been measured are represented.

Here, we present a consolidated set of phenotypic data from genetic trials of *Fagus sylvatica* across Europe compiled by the COST Action E52 (2006–2010). There have been several efforts to make use of phenotypic data from forestry trials of various species at the European level e.g. to compile metadata and to produce standardised protocols (http://www.trees4future.eu/ and http://www.trees4future.eu/transnational-accesses/treebreedex.html). However, raw data is seldom published, and initiatives to compile old genetic trials tend only to be promoted at the national level; e.g. GENFORED in Spain (www.genfored.es) and PLANTACOMP in France^5^. The BeechCOSTe52 dataset (Data Citation 1) provides a high density map of phenotypic data from 38 genetic trials across Europe, even including trials established outside the range of the species. The wide coverage provided by this dataset makes it a singular resource that can help us to understand the entire gradient of climatic tolerance of European beech (Fig. 1). Likewise, the origin of the 217 provenances planted covers the entire range of the species distribution, which is important for understanding the overall adaptive capacity and the tolerance limits of the species (Fig. 1).

Genetic trials have historically been used in forestry to test which reproductive material to select (http://www.euforgen.org), but the utility of genetic trials in general and of this database in particular go beyond this traditional use^6^. The most immediate use of the BeechCOSTe52 database is in understanding of trade-offs between traits from an ecological perspective. Comparable trials over a larger geographical areas covering the range limits of a species can be helpful in assessing local adaptation in populations of trees that have grown nearby during the recent past. They can also be useful for testing the fitness of range-edge populations or those derived from isolated refuges during the last glaciation, which may hold valuable traits that can confer fitness benefits under particular circumstances. Over small geographical areas, the effects of local environmental conditions and past management can be tested and pave the way to guide new innovative management practises involving assisted gene flow and the translocation of populations to compensate lower productivity with higher survival when mitigating climate change and the socio-economic consequences of planting exotic versus local provenances.

A complete network of genetic trials is also of interest for the conservation of genetic resources, in particular those from marginal populations which can hold higher genetic variation than those populations at the core of a distribution^7^. Finally, we hope that this database will encourage researchers and foresters to compile and publish genetic trial data of other species, since databases such as this are an invaluable resource helping us understand the capacity of forests to adapt and acclimatise to climate change.

## Methods

### Tree plantation in genetic trials

The international series of genetic trials presented in the BeechCOSTe52 database were established in two different series (years sown/trial planted: 1993/95 and 1996/98) to study genetic variation of European beech across its distribution range^8^. The planning of the international series of beech trials was principally coordinated by researchers from the Institute of Forest Genetics in Grosshansdorf, Germany, including Mirko Liesebach, Georg von Wuehlisch and Hans Muhs, in liaison with the international trial-site holders, and supported by the Concerted Action of the Commission of the European Communities (AAAIR3 Programme, Grant No. CT94-2091). Previous plantations of genetic trials of beech exist but mostly with local provenances, which precludes the study of genetic variation across the species range. The trials were planted in three complete blocks, each block containing all of the provenances planted at each trial site. In total 150 seedlings (two-year old) of each provenance were planted in randomised groups of 50 seedlings within each block, using a spacing of 1-×-2 m. Border rows of a local provenance were used to reduce edge effects. More details of the planning, design and planting of the provenance trials are given in a document presented at the IUFRO meetings on beech^8^.

### Phenotypic traits measurement

**Figure 2:**
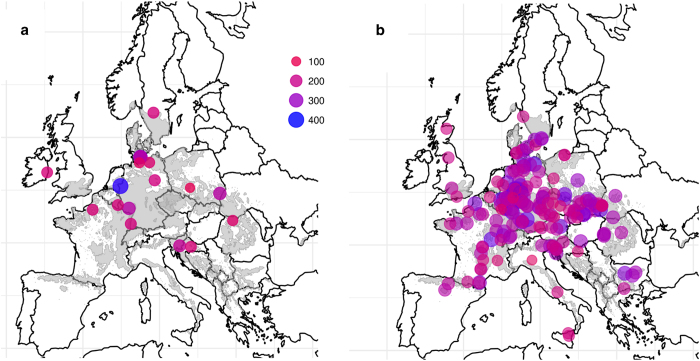
Tree height averaged by genetic trial and provenance tested, plotted at their corresponding location across Europe. **(a)** Tree height (cm) averaged by trial site (maximum tree height 400 cm) and by **(b)** provenance (tree height ranged from 100 to 300 cm) of 9 years old trees in both series (seedlings were planted at two years of age). The distribution of *Fagus sylvatica* is reproduced in grey from EUFORGEN http://www.euforgen.org/distribution-maps/. Plotted symbols represent tree height in a size and colour scale running from 100 cm (small red circles) to 400 cm (big blue circles) high.

Phenotypic trait measurements included tree height, basal diameter, diameter at breast height, mortality, bud burst phenology in spring and autumn leaf senescence, and in some cases the causes of damage related to mortality. Trait data was collected on an individual-trees basis in consecutive years following plantation, including measurements from 2-to-15 year-old trees, thus providing information through different ontogenic stages. We give an example of how the database can be plotted and further analysed according to trait and ontogenic stage (Fig. 2).

### Data compilation

A succession of national and international projects have allowed the collection of data from the trial sites (Additional Information.zip, Data Citation 1 and 1^st^ Meeting^9^). In 2004, various preliminary databases of individual French, German and Italian provenances were gathered during the DYNABEECH project (Additional Information.zip, Data Citation 1).

Later on, a European Concerted Research Action designated as COST Action E52 "Evaluation of Beech Genetic Resources for Sustainable Forestry" came into force in 2006, running until 2010, in order to consolidate and interpret the data collected from the two series of beech trials set up in 1995 and 1998, and to bring together the participants from countries that were managing trials to pool their expertise. The stated aim of the COST Action was “to make predictions of the future distribution range of beech forest ecosystems under the assumption of certain scenarios of climate change, based on the analysis of the reaction pattern of European beech populations”.

An introductory COST Action E52 Working Group and Management Committee meeting took place in Zvolen (Slovakia 2006), with plenary meetings during the subsequent three years as well as various Working Group meetings^10^. Some partial analyses were done during this period, considering one^11–18^ or several trials^19–23^.

One striking quality of the BeechCOSTe52 database presented here is that it has been made possible through the coordinated effort of researchers and foresters from many countries across Europe, who have managed and maintained the trial sites over an extended period of time and have altruistically contributed to the efforts of data collection and coordination over a long time period. The compilation of the phenotypic information from all the genetic trials included in the BeechCOSTe52 database only refers to trials and provenances in which traits have been measured repeatedly (Table 1). The database includes 862,095 trait records measured in 38 trial sites and a total of 217 provenances varying from 13 to 101 among trial sites. We kept only traits measured in at least six trial sites to assure a good coverage of the distribution of the species. Measurements are heterogeneously repeated over years and trees, from 1995 to 2008.

### Code availability

R code to merge phenotypic trait measurements, geographical coordinates of the trials and geographical coordinates of the provenance locations is included (merge-trial-prov-coords_individualmeasurements.R). Mapping phenotypic traits by provenances and trials as shown in Figs. 1 and 2 can be reproduced using the following scripts: Hplot.R & Plot_Trial-Prov_locations.R. Likewise, individual data by trial and trait can be extracted from the global database using the specific script Subset_by_trial.R and Subset_by_trait.R, respectively.

## Data Records

The database is structured in three independent files that can be merged using the code identifier for the provenance and the code combining the trial and provenance codes ("Trial" and "ID_ProvCode", respectively). These data files together with the metadata descriptor document are available at Zenodo data repository (BeechCOSTe52_metadata_descriptor.docx, Data Citation 1). The first file contains individual measurements of phenotypic traits (Fsylvatica.csv, Data Citation 1): height, basal diameter, diameter at breast height, mortality, spring phenology, autumn leaf phenology, and associated damage causing to tree mortality. The second file contains the description of the trials including their geographical locations (Trials_coord.csv, Data Citation 1) and the third file describes the locations of the seed sources (Prov_coord.csv, Data Citation 1).

## Technical Validation

The database has been checked for consistency at different stages by various researchers. During the COST Action E52 (2006 to 2010) raw data were submitted by each country and harmonized in electronic format for the first time. Results from the individual trial sites were sent to Georg von Wuehlisch until 2008. In 2008 and 2009 this information was assimilated into electronic form and standardised by Diana Barba and T. Matthew Robson. At that time, it was decided to have one database by genetic trial, harmonised among trials and checked for consistency. The geographical locations, names and identities of trial sites and provenances were rechecked, and the management and biogeographical history of provenances considered. In a workshop (19^th^-23^rd^ January 2009) the data evaluation working group met in Valsain, Spain, where the database was checked for completeness and consistency of data collected by the members of the COST Action E52. Then, members of the COST Action E52 had one year to present their findings prior to the 2010 final Cost Action E52 meeting in Burgos, where various studies of the provenance trials were presented, and later published in the Proceedings of the Meeting^21^ (Additional Information.zip, Data Citation 1).

The studies reported that the data gathered expose high variability in fitness-related traits such a height, both among provenances and among trials^24^. Likewise, there was large variation in the timing of bud burst and leaf flush in spring, when data collected using different scales among sites were harmonised and considered both in terms of day-of-the-year and degree-hours of forcing prior to bud burst^25^.

Finally, in 2017, the originally-compiled trial-site database was checked again for consistency of entries and fields in R by Marta Benito Garzón prior to harmonization of all the trial sites into the single phenotypic database presented here. Outliers and errors in the database were checked by calculating descriptive statistics of quantitative traits for each trial averaged by provenance.

## Additional information

**How to cite this article**: Robson, T. M. *et al.* Phenotypic trait variation measured on European genetic trials of *Fagus sylvatica* L. *Sci. Data* 5:180149 doi: 10.1038/sdata.2018.149 (2018).

**Publisher**’**s note**: Springer Nature remains neutral with regard to jurisdictional claims in published maps and institutional affiliations.

## Supplementary Material



## Figures and Tables

**Table 1:** Summary table showing the information contained in the dataset by trial site.

**TRIAL CODE**	**TRIAL NAME (COUNTRY)**	**PROV**	**Height**	**DBH**	**BasalD**	**Mort**	**SP**	**AP**	**D**
BU1901	Schädtbek (DE)	100	45,727	32,830	0	0	4,396	7,095	0
BU1902	Wesel (DE)	101	6,036	0	0	0	0	0	0
BU1904	Burguete, Navarra (ES)	100	10,520	0	10,521	0	0	0	0
BU1905	Vrchdobroč (SK)	97	4,096	0	0	0	8,308	1,951	0
BU1908	Otterup (DK)	49	19,937	0	0	0	2,935	0	12,310
BU1909	F.d. Lyons (FR)	50	17,309	4,413	0	7,232	16,850	0	0
BU1910	Brasimone (IT)	49	4,059	4,056	0	0	0	0	0
BU1913	Loehlitz (DE)	48	0	0	0	7,200	0	0	0
BU1914	Pelhrimov (CZ)	49	5,667	0	0	7,350	0	0	5,625
BU1915	Oleszyce (PL)	49	21,667	24,584	0	0	9,511	4,171	0
BU1917	Braşov (RO)	44	6,892	1,240	1,273	0	10,421	0	0
BU1921	Ranna (SE)	36	4,789	0	0	5,400	1,413	0	21,600
BU1922	Louchelt (LU)	49	11,271	1,543	0	0	3,795	0	2,349
BU1923	Baia Mare (RO)	26	4,679	777	1,560	0	0	0	0
BU2001	Schädtbek(DE)	46	14,385	4,639	0	0	11,148	0	11,100
BU2002	Gädebehn (DE)	17	4,009	811	0	0	0	0	0
BU2004	Laragh (IE)	34	3,652	0	0	0	3,103	0	0
BU2005	Little Wittenham (UK)	25	1,060	986	0	0	8,863	0	0
BU2006	Harre (BE)	34	2,907	0	0	0	0	0	0
BU2007	Bosenbach (DE)	21	10,074	3,059	0	0	0	0	0
BU2008	Oostereng (NL)	31	6,791	0	0	0	6,745	0	0
BU2009	Lisbjerg (DK)	34	5,738	0	0	4,054	5,362	0	1,226
BU2010	Trolleholm (SE)	33	3,690	0	0	4,851	2,914	0	64
BU2011	Nedlitz-1 (DE)	33	4,068	0	0	0	0	0	0
BU2012	Straža (SI)	39	26,570	5,718	0	0	8,845	0	2,140
BU2014	Unieszow (PL)	34	1,084	313	0	0	2,807	0	0
BU2016	Kutina (HR)	19	13,246	0	0	0	0	0	0
BU2017	Alunis (RO)	32	1,890	1,773	0	0	0	0	0
BU2018	Poiana Fl (RO)	32	2,880	926	960	0	10,607	0	0
BU2019	Jíloviště (CZ)	31	5,252	1,271	0	0	8,646	2,893	0
BU2020	Tále, Mláčik (SK)	32	12,846	2,346	3,093	0	65,080	6,231	6,231
BU2021	Valle de Mena (ES)	33	0	0	0	1,940	3,598	0	0
BU2022	Liliental (DE)	20	4,026	0	0	1,500	0		1,500
BU2023	Hahnengrün (DE)	30	14,047	6,598	0	0	20,994	12,040	0
BU2024	Pazuengos, La Rioja (ES)	29	3,641	0	3,644	4,330	3,679	0	0
BU2025	Nedlitz-2 (DE)	13	4,731	1,057	0	0	0	0	0
BU2026	Louschelt (LU)	28	11,213	2,198	0	0	0	0	0
BU2028	Rantzau (DE)	18	2,035	555	0	0	0	0	0
TOTAL=	**862,095**	**217**	**333,677**	**101,693**	**21,051**	**43,857**	**220,020**	**77,652**	**64,145**
The summary information refers to trait measurements in one or several years and can include several trait measurements per tree. PROV=number of provenances planted. Height=number of height measurements. DBH=number of diameter at breast height measurements. BasalD=number of basal diameter measurements. Mort=number of dead trees. SP=number of bud burst measurements. AP=number of records of autumn leaf discolouration. D=number of damage measurements. Zero values indicate no data available.									

## References

[d1] Zenodo Robson T. M. *et al.* 2018 https://doi.org/10.5281/zenodo.1240931

